# Effects of calcifediol supplementation on markers of chronic kidney disease‐mineral and bone disorder in dogs with chronic kidney disease

**DOI:** 10.1111/jvim.15949

**Published:** 2020-10-31

**Authors:** Valerie J. Parker, Adam J. Rudinsky, Jason A. Benedict, Azadeh Beizaei, Dennis J. Chew

**Affiliations:** ^1^ Veterinary Clinical Sciences The Ohio State University Veterinary Medical Center Columbus Ohio USA; ^2^ Center for Biostatistics, Department of Biomedical Informatics The Ohio State University College of Medicine Columbus Ohio USA; ^3^ EirGen Pharma LTD, R&D Center IDA Business and Technology Park Waterford Ireland

**Keywords:** 25‐hydroxyvitamin D, calcitriol, fibroblast growth factor‐23, fractional excretion, parathyroid hormone, urine calcium‐to‐creatinine ratio

## Abstract

**Background:**

Chronic kidney disease‐mineral and bone disorder (CKD‐MBD) in dogs is associated with hypovitaminosis D, increased parathyroid hormone (PTH), and increased fibroblast growth factor‐23 (FGF‐23) concentrations. Best practice for vitamin D metabolite supplementation in CKD‐MBD remains unknown.

**Objective:**

To provide an extended‐release calcifediol supplement to dogs with CKD and to measure its effects on variables indicative of CKD‐MBD.

**Animals:**

Ten dogs with International Renal Interest Society stages 2 and 3 CKD.

**Methods:**

In a prospective study, dogs received a calcifediol supplement for 84 days. Serum 25‐hydroxyvitamin D (25[OH]D), 1,25‐dihydroxyvitamin D (1,25[OH]_2_D), 24,25‐dihydroxyvitamin D (24,25[OH]_2_D), creatinine, calcium, phosphorus, PTH, plasma FGF‐23 concentrations, and urine profiles were measured monthly during supplementation. Urine calcium to creatinine (UCa/Cr) ratios and fractional excretion of calcium, phosphorus, and sodium were determined.

**Results:**

All serum vitamin D metabolite concentrations increased significantly by day 84 (*P* < .001): [25(OH)D (median 249.9 ng/mL; range, 149.7‐469.9 ng/mL) compared to baseline (median 50.2 ng/mL; range, 31.3‐66.0 ng/mL); 1,25(OH)_2_D (median 66.1 pg/mL; range, 56.9‐88.1 pg/mL) compared to baseline (median 37.3 pg/mL; range, 29.3‐56.7 pg/mL); 24,25(OH)_2_D (median 81.4 ng/mL; range, 22.1‐151.7 ng/mL) compared to baseline (median 15.4 ng/mL; range, 6.9‐40.6 ng/mL)]. There were no significant differences in calcium, phosphorus, PTH concentrations, UCa/Cr or fractional excretion of calcium. No dog developed ionized hypercalcemia. Plasma FGF‐23 concentrations increased by day 84 (median 1219 pg/mL; range, 229‐8824 pg/mL) compared to baseline (median 798 pg/mL; range, 103‐4.145 pg/mL) (*P* < .01).

**Conclusions and Clinical Importance:**

Calcifediol supplementation for 84 days was well‐tolerated in dogs with IRIS stages 2 and 3 CKD. It remains to be determined how long‐term supplementation would affect CKD progression and QOL.

**Abbreviations**
1,25(OH)_2_D: 1,25‐dihydroxyvitamin D
24,25(OH)_2_D: 24,25‐dihydroxyvitamin D
24(OH)ase: 24‐hydroxylase
25(OH)D: 25‐hydroxyvitamin D
CaPP: calcium × phosphorus product
CKD: chronic kidney disease
CKD‐MBD: chronic kidney disease‐mineral bone disorder
FE: fractional excretion
FGF‐23: fibroblast growth factor‐23
iCa: ionized calcium
IRIS: International Renal Interest Society
PTH: parathyroid hormone
QOL: quality of life
tCa: total calcium
UCa/Cr: urine calcium to creatinine
UPC: urine protein to creatinine
USG: urine specific gravity

## 1 INTRODUCTION

Chronic kidney disease (CKD) in dogs is a condition characterized by progressive loss of function, with a prevalence of up to 25% of dogs.^1^, ^2^, ^3^ One major consequence of CKD is the development of CKD‐mineral and bone disorder (CKD‐MBD). This is characterized by altered calcium‐phosphorus homeostasis, decreased concentrations of vitamin D metabolites, specifically 25‐hydroxyvitamin D [25(OH)D], 1,25‐dihydroxyvitamin D [1,25(OH)_2_D; calcitriol], and 24,25‐dihydroxyvitamin D [24,25(OH)_2_D], and increased concentrations of parathyroid hormone (PTH) and fibroblast growth factor‐23 (FGF‐23).^4^ These derangements correlate with International Renal Interest Society (IRIS) stage.^5^, ^6^ In people with CKD, hypovitaminosis D and increased serum PTH and FGF‐23 concentrations are associated with disease progression and decreased survival.^7^, ^8^, ^9^ In dogs with CKD, increased serum phosphorus, calcium × phosphorus product (CaPP), as well as FGF‐23 concentrations are negatively correlated with survival.^10^, ^11^

In human medicine, management of CKD‐MBD utilizes an integrated approach, including dietary phosphate restriction and oral phosphate binders (to reduce intestinal phosphate absorption), calcimimetics (to lower PTH by enhancing activation of the calcium‐sensing receptor), and vitamin D sterols (to increase intestinal calcium absorption and to inhibit PTH genomic synthesis and secretion).^12^ There is no widely accepted best practice for how to supplement vitamin D. Per the 2017 Kidney Disease Improving Global Outcomes report, vitamin D_3_ (ie, cholecalciferol) and D_2_ (ie, ergocalciferol) therapy remains unproven. High doses of oral vitamin D_3_ or vitamin D_2_ supplementation often do not reliably increase 25(OH)D concentrations in people.^13^, ^14^ Prophylactic calcitriol therapy is no longer recommended due to risk of hypercalcemia.^15^ While there was not enough data to specifically comment on the use of calcifediol [25(OH)D], some recent literature suggests that calcifediol is safe and effective in treating hyperparathyroidism associated with hypovitaminosis D in people with CKD.^16^, ^17^

In veterinary medicine, there is similar emphasis on controlling oral phosphate intake and serum phosphorus concentrations, but there has been little research specifically into how to best utilize vitamin D supplementation. In healthy dogs, high doses of cholecalciferol (ie, vitamin D_3_, the precursor to 25(OH)D via hepatic metabolism) are required to affect serum 25(OH)D concentrations.^18^, ^19^ Calcitriol has a narrow therapeutic window and there is risk of hypercalcemia and an increased CaPP if hyperphosphatemia is not adequately controlled. Calcifediol [25(OH)D] has been shown to significantly increase serum 25(OH)D concentrations in healthy dogs,^20^ but it has not been evaluated in dogs with CKD.

The primary goal of this pilot study was to assess the effects of calcifediol supplementation on markers of CKD‐MBD in dogs with CKD. Our hypotheses were that calcifidiol supplementation would increase serum concentrations of vitamin D metabolites and decrease PTH concentrations with minimal effect on calcium‐phosphorus homeostasis.

## 2 MATERIALS AND METHODS

### 2.1 Case selection criteria

Ten client‐owned adult dogs with IRIS stages 2 and 3 CKD were prospectively enrolled, from September 2017 through June 2018, according to the 2017 IRIS Staging of CKD guidelines. A diagnosis of CKD was made based on the presence of at least 2 episodes, over at least 3 months, of minimally concentrated urine (urine specific gravity [USG] < 1.030) with azotemia in the absence of other diseases likely to cause polyuria or polydipsia. Most dogs (n = 6) had ultrasonographic evidence of CKD (eg, decreased corticomedullary distinction). One dog had previously had a renal biopsy documenting membranoproliferative glomerulonephritis (stable, nonproteinuric at time of enrolment). Dogs were included if they were at least 1 year of age and weighed ≥10 kg. Each dog was initially screened to ensure that it had an ionized calcium (iCa) concentration ≤5.8 mg/dL.

Dogs diagnosed with acute kidney injury or suspected acute exacerbation of CKD were excluded. Dogs diagnosed with primary hyperparathyroidism or primary hypoparathyroidism, protein‐losing enteropathy, or neoplasia were excluded. Dogs with a urine protein to creatinine (UPC) ratio > 2.0 or a urinary tract infection diagnosed via urine culture were excluded. Dogs receiving corticosteroids or calcitriol therapy were excluded. The study was approved by The Ohio State University Institutional Animal Care and Use Committee, and all owners signed a consent form before dogs were enrolled in the study.

### 2.2 Study design

Dogs were screened initially to ensure study inclusion and exclusion criteria were met. Dogs then returned within a 2‐week timeframe to complete enrolment and to ensure that their serum creatinine concentrations were stable. Dogs were prescribed an extended‐release 25(OH)D medication approved for use in humans with CKD (Rayaldee, OPKO Renal Health, Miami, Florida). Dosage was based on body weight to provide approximately 2.0 μg/kg/day. This dose was extrapolated from data in healthy dogs.^20^ Premade capsules were available in 30 and 60‐μg capsules, and dogs received either 1 or 2 capsules per day, in the evening, with food. Dogs received the supplement for 84 days and were rechecked every 28 days. After preliminary review of data from the first 4 dogs enrolled, based on the degree and rapid nature of the rise in serum 25(OH)D concentrations, calcifediol dosing was modified to be provided on a Monday‐Wednesday‐Friday (M‐W‐F) schedule.

At baseline, and for the monthly rechecks while dogs were receiving the calcifediol supplement, a complete physical examination, including body weight, body condition score and muscle condition score, as well as funduscopic examination, was performed. A blood pressure was measured via Doppler (average of 5 measurements). Blood and urine were collected for CBC, serum chemistry profile, serum iCa, urinalysis, UPC, urine calcium to creatinine (UCa/Cr) ratio, fractional excretion (FE) of calcium, phosphorus and sodium, and 25(OH)D. These samples were processed for analysis within 30 minutes. Extra serum and plasma was frozen at −80°C for PTH, 1,25(OH)_2_D, 24,25(OH)_2_D, and FGF‐23 measurements. Study dogs were fasted overnight for all visits, and each visit was completed at the same time of day, usually in the morning.

After 84 days of calcifediol supplementation, owners were instructed to discontinue the supplement. Vitamin D metabolites, PTH, and FGF‐23 concentrations were measured 28 and 56 days later. CBC and chemistry profiles were obtained 56 days after discontinuation.

### 2.3 Serum and urine profiles

The serum chemistry and urine profiles, including concentrations of creatinine, total calcium (tCa), and phosphorus, were performed using a Roche Cobas 6000 c501 chemistry analyzer. Samples used for serum iCa concentrations were handled anaerobically and were measured within 30 minutes using a Stat Profile pHOx Ultra analyzer. Fractional excretion of urine calcium and phosphorus were calculated by this equation: [serum creatinine]/[urine creatinine] divided by [serum electrolyte]/[urine electrolyte] × 100 or [urine electrolyte] × [serum creatinine] divided by [urine creatinine] × [serum electrolyte] × 100.^21^

### 2.4 Vitamin D analysis

Serum 25(OH)D, 1,25(OH)_2_D, and 24,25(OH)_2_D concentrations were measured by liquid chromatography‐mass spectrometry by a Vitamin D External Quality Assessment Scheme (DEQUAS)‐certified laboratory (Heartland Assays, Inc, Ames, Iowa).

### 2.5 PTH and FGF‐23 analysis

Serum whole PTH concentrations were measured with an immunoradiometric assay utilizing a polyclonal 1‐84 PTH antibody (Michigan State University Diagnostic Center for Population and Animal Health, East Lansing, Michigan). Interassay coefficient of variation is reported to be 10%, intra‐assay coefficient of variation is reported to be 3%, and functional sensitivity is reported to be 0.3 pmol/L for this assay. Plasma FGF‐23 concentrations were measured using a human‐specific ELISA (Kainos FGF‐23 ELISA, Japan), previously validated for measurement in dogs.^22^

### 2.6 Quality of life questionnaire

Owners filled out a quality of life (QOL) questionnaire at screening, baseline and every month for 3 months while receiving the calcifediol supplement (Appendix). This questionnaire was modified from 2 previously‐validated questionnaires for dogs with cancer^23^ and cats with CKD.^24^ Owners rated 14 parameters related to their dogs' (a) happiness, (b) mental status, (c) appetite and gastrointestinal health, (d) mobility and recreation, (e) general health. Each question could receive a score of 1 to 5, with lower scores corresponding to lower QOL and higher scores corresponding to higher QOL. The highest score a dog could receive was 70.

### 2.7 Data analysis

Descriptive statistics are reported for all collection times. Multilevel models with random intercepts for each dog were fit to analyze differences in outcomes of interest over time induced from dosing. To test for associations between concentrations of 25(OH)D and both serum tCa and serum phosphorus concentrations, multilevel models with random intercepts and slopes for each dog were fit. Similarly, to test for associations between QOL scores and both serum creatinine and serum 25(OH)D concentrations, multilevel models with random intercepts and slopes for each dog were fit. A multilevel model with a random intercept and slope for each dog was also used to test for an association between FGF‐23 concentrations and FE of phosphorus concentrations. All confidence intervals are 2‐sided and presented at their nominal level. *P*‐values ≤.05 were considered statistically significant. All analyses were conducted in Stata, version 15.1 (StataCorp. 2017. *Stata Statistical Software: Release 15*. College Station, Texas: StataCorp LLC).

## 3 RESULTS

A total of 11 dogs were screened for inclusion into the study. A single dog was excluded from the study based on a positive urine culture and diagnosis of a clinical urinary tract infection. The remaining 10 dogs met all inclusion and exclusion criteria and were diagnosed with IRIS stage 2 and 3 CKD. At the time of screening, median age of the 10 enrolled dogs was 8.5 years (range, 1‐13 years). Breeds represented were mixed breed dog (n = 3), German shepherd dog (n = 2), and 1 each of the following: American pit bull terrier, Bernese mountain dog, Collie, Golden retriever, Labrador retriever. Median body weight was 26.7 kg (range, 15.3‐41.2 kg). Median body condition score was 6/9 on a 9‐point scoring system (range, 5‐8). Muscle condition was noted to be normal in 6/10 dogs and mild atrophy was noted in 4/10 dogs.

### 3.1 IRIS stages and substages

Pertinent laboratory variables are listed in Table 1. At baseline, dogs were classified as either stage 2 (n = 4) or stage 3 (n = 6). Median serum creatinine was 2.3 mg/dL (range, 1.7‐4.5 mg/dL). Median USG was 1.015 (range, 1.005‐1.020). Median UPC was 0.27 (range, 0.06‐1.87). Based on IRIS substages, dogs were classified as nonproteinuric (n = 5), borderline proteinuric (n = 3) or proteinuric (n = 2). All dogs had a negative urine culture. Median blood pressure was 133 mm Hg (range, 118‐153 mm Hg). Each dog received a blood pressure substage: normotensive (n = 6), prehypertensive (n = 4) (Table 1).

**TABLE 1:** Laboratory variables of CKD dogs at baseline, while receiving calcifediol supplementation (through day 84), and after discontinuation of calcifediol supplementation.

Laboratory variable and reference range	Baseline (n = 10)	Day 28 (n = 10)	Day 56 (n = 10)	Day 84 (n = 10)	Day 112 (n = 9)	Day 140 (n = 9)
Hematocrit (40%‐59%)	39 (30‐44)	NM	NM	NM	NM	37 (25‐41)
BUN (5‐20 mg/dL)*	42 (9‐73)	39 (9‐79)	38 (13‐93)	43 (12‐85)	NM	47 (14‐93)
Creatinine (0.6‐1.6 mg/dL)	2.3 (1.7‐4.5)	2.5 (1.4‐5.3)	2.6 (1.5‐6.1)	2.5 (1.6‐4.9)	NM	3.4 (1.6‐6.6)
Phosphorus (3.2‐8.1 mg/dL)*	3.8 (2.7‐6.5)	4.1 (2.6‐6.6)	4.2 (2.5‐7.0)	4.4 (2.7‐6.9)	NM	4.6 (3.1‐8.5)
Total calcium (9.3‐11.6 mg/dL)*	10.6 (9.6‐11.9)	10.8 (9.2‐12.0)	10.8 (9.3‐11.7)	11.3 (9.5‐12.5)	NM	11.2 (9.4‐13.2)
Ionized calcium (4.9‐5.8 mg/dL)*	5.06 (4.79‐5.37)	5.09 (4.71‐5.49)	5.10 (4.68‐5.41)	5.07 (4.70‐5.71	NM	NM
Ca × P product (mg^2^/dL^2^)*	39.5 (25.9‐77.4)	43.4 (23.9‐29.2)	42.5 (25.0‐80.5)	48.0 (25.7‐86.3)	NM	50.3 (19.1‐101.2)
USG	1.015 (1.005–1.020)	NM	NM	NM	NM	NM
UPC	0.27 (0.06–1.87)	NM	NM	NM	NM	NM
Urine calcium : creatinine (UCa : Cr) ratio	0.01 (0.00‐0.07)	0.01 (0.01‐0.12)	0.01 (0.00‐0.13)	0.02 (0.00‐0.11)	NM	NM
Fractional excretion (FE %) for total calcium	0.18 (0.11‐1.72)	0.38 (0.13‐3.46)	0.35 (0.09‐4.30)	0.51 (0.03‐4.08)	NM	NM
Fractional excretion (FE %) for ionized calcium	0.38 (0.20‐3.89)	0.75 (0.26‐8.18)	0.76 (0.17‐9.69)	1.16 (0.07‐8.93)	NM	NM
Fractional excretion (FE %) for phosphorus*	25.4 (8.2‐39.7)	18.3 (3.0‐31.8)	19.2 (2.9‐52.4)	19.7 (1.1‐52.9)	NM	NM
Parathyroid hormone (0.5‐5.8 pmol/L)	3.9 (1.4‐20.1)	3.2 (1.0‐21.4)	3.7 (0.9‐43.7)	2.8 (0.5‐43.2)	5.2 (0.7‐90.1)	3.1 (2.2‐72.4)
Fibroblast growth factor‐23 (pg/mL)	798 (103‐4145)	732 (155‐4288)	951 (115‐9725)	1219 (229‐8824)	1809 (403‐30 085)	1555 (234‐37,002)
Blood pressure (mm Hg)	133 (118–153)	129 (110‐151)	136 (106‐173)	129 (118‐146)	NM	NM

*Note*: Data are presented as medians and ranges. Normally distributed variables are noted with an asterisk (*).

Abbreviation: NM, not measured.

### 3.2 Vitamin D supplementation and vitamin D metabolites

**FIGURE 1:**
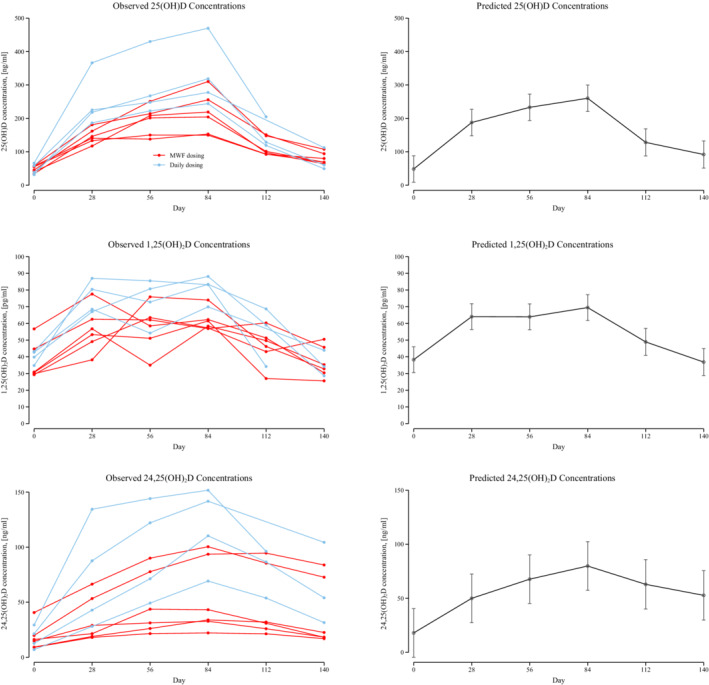
Serum concentrations of vitamin D metabolites [25(OH)D, 1,25(OH)_2_D, and 24,25(OH)_2_D] at baseline, during 84 days of extended‐release calcifediol supplementation, and after discontinuation of calcifediol supplementation. Dogs receiving daily dosing are graphed in blue and dogs receiving intermittent dosing on Monday, Wednesday, and Friday each week (M‐W‐F dosing) are graphed in red. Line plot with means and 95% confidence intervals of predicted vitamin D metabolites [25(OH)D, 1,25(OH)_2_D, and 24,25(OH)_2_D] concentrations over time based on multilevel models from all 10 dogs in this study

**FIGURE 2:**
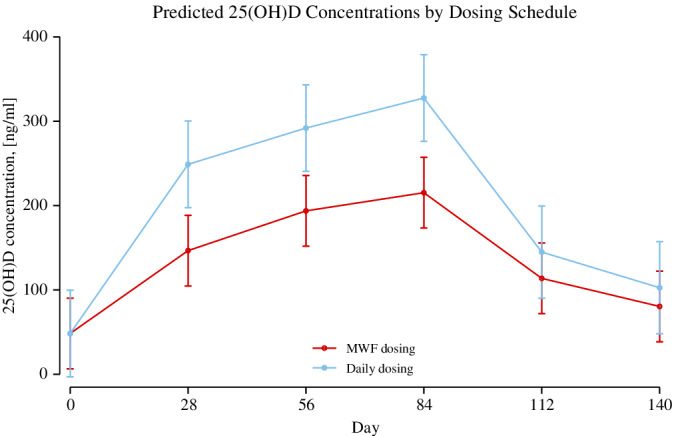
Predicted serum 25(OH)D concentrations over time based on a multilevel model from data from the 10 dogs in this study receiving daily dosing (n = 4) and intermittent dosing on Monday, Wednesday, and Friday each week (M‐W‐F dosing) (n = 6). Dogs receiving daily dosing are graphed in blue, and dogs receiving M‐W‐F dosing are graphed in red. Data is shown for dogs at baseline, during 84 days of extended‐release calcifediol supplementation, and after discontinuation of calcifediol supplementation. Line plots with means and 95% confidence intervals shown

Serum concentrations of 25(OH)D, 1,25(OH)_2_D and 24,25(OH)_2_D at baseline and each monthly recheck are presented in Table 2. The median dose of calcifediol provided to dogs was 2.0 μg/kg/dose (range, 1.6‐2.7 μg/kg/dose). There was evidence that concentrations of 25(OH)D, 1,25(OH)_2_D, and 24,25(OH)_2_D were increased after supplementation at months 1 through 3 compared to baseline (*P* < .001 for all comparisons, Figure 1). Figure 2 illustrates the differences in serum 25(OH)D concentration over time with different calcifediol dosing by comparing daily dosing (n = 4) to M‐W‐F dosing (n = 6). Daily dosing increased the concentration of 25(OH)D levels compared to M‐W‐F dosing (*P* < .001 for months 1‐3, Figure 2). In comparison to peak concentrations at day 84, serum 25(OH)D decreased at day 112 by an average of 132 ng/mL (95% CI: 98, 167; *P* < .001) and an average of 168 ng/mL at day 140 (95% CI: 134, 203; *P* < .001). Serum 1,25(OH)_2_D decreased at day 112 compared to day 84 by an average of 21 pg/mL (95% CI: 11, 30; *P* < .001) and decreased by an average of 33 pg/mL (95% CI: 23, 43; *P* < .001) at day 140 compared to day 84. Serum 24,25(OH)_2_D concentrations decreased at day 112 compared to peak concentrations at day 84 with an average decrease of 17 ng/mL (95% CI: 1, 32; *P* = .03) as well as at day 140 compared to day 84 with an average of 27 ng/mL (95% CI: 12, 43; *P* < .001).

**TABLE 2:** Serum vitamin D metabolite concentrations [25(OH)D, 1,25(OH)_2_D, and 24,25(OH)_2_D] at baseline, during 84 days of extended‐release calcifediol supplementation, and after discontinuation of calcifediol supplementation.

Vitamin D metabolite	Baseline (n = 10)	Day 28 (n = 10)	Day 56 (n = 10)	Day 84 (n = 10)	Day 112 (n = 9)	Day 140 (n = 9)
25‐hydroxyvitamin D (ng/mL)	50.2 (31.3–66.0)	171.4 (117.3‐366.3)	218.3 (137.9‐430.0)	249.9 (149.7‐469.9)	119.1 (92.4‐204.7)	68.7 (49.4‐112.3)
1,25‐dihydroxyvitamin D (pg/mL)*	37.3 (29.3‐56.7)	64.8 (38.2‐87.0)	62.8 (35.0‐85.5)	66.1 (56.9‐88.1)	49.7 (27.0‐68.7)	34.1 (25.6‐50.5)
24,25‐dihydroxyvitamin D (ng/mL)	15.4 (6.9‐40.6)	35.9 (18.0‐134.4)	60.3 (21.5‐144.1)	81.4 (22.1‐151.7)	53.8 (21.3‐96.2)	31.5 (16.9‐104.4)
25(OH)D : 24,25(OH)_2_D ratio	2.9 (1.4‐6.5)	4.6 (2.0‐10.1)	3.8 (1.7‐10.0)	3.9 (1.5‐11.6)	2.4 (1.1‐7.1)	1.9 (0.8‐5.6)

*Note*: Data are presented as medians and ranges. Normally distributed variables are noted with an asterisk (*).

### 3.3 Calcium and phosphorus

**FIGURE 3:**
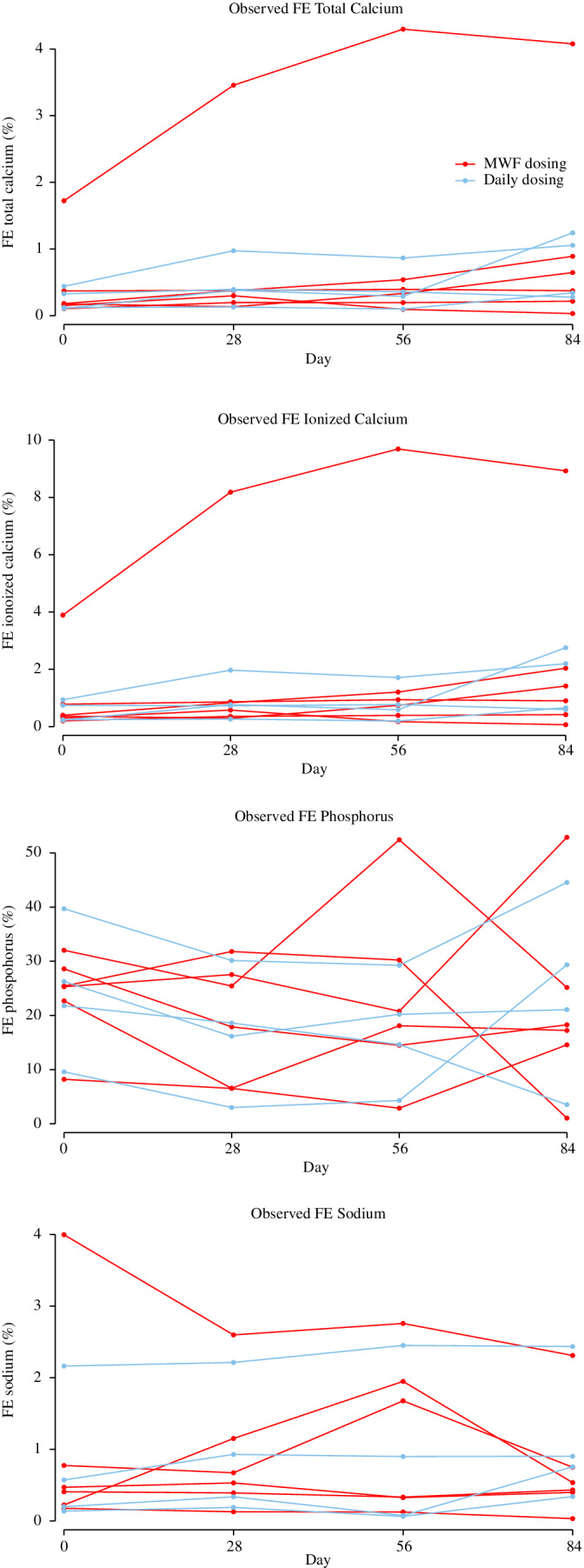
Fractional excretion (FE %) of total calcium, ionized calcium, phosphorus, and sodium at baseline and during 84 days of extended‐release calcifediol supplementation. Each line plot represented observed values from an individual dog. Dogs receiving daily dosing are graphed in blue, and dogs receiving intermittent dosing on Monday, Wednesday, and Friday each week (M‐W‐F dosing) are graphed in red

Serum tCa, iCa, and phosphorus concentrations and UCa/Cr ratios were monitored during the study to evaluate for hypercalcemia, hyperphosphatemia, or increased calciuresis associated with the calcifediol supplement. The serum tCa concentrations are nearly identical to those of serum iCa concentrations, so only tCa is reported. tCa increased by 0.54% (95% CI: 0.21%, 0.88%; *P* = .002) at day 84. One dog appeared to respond more than all others, and, when removed, the tCa concentration increased by 0.34% (95% CI: 0.16%, 0.53%; *P* = .02) at day 84. There were no observed differences in serum phosphorus or sodium concentrations (*P* = .62 and .75, respectively) at any time over the course of supplementation (Figure 3). No incidences of ionized hypercalcemia (ie, > 5.8 mg/dL) were noted throughout the study. Four dogs demonstrated a total hypercalcemia during the course of the study; of these 4 dogs, 1 dog received daily dosing and 3 dogs received M‐W‐F dosing. Three dogs were normocalcemic at baseline and developed hypercalcemia either at day 28 (n = 1) or by day 84 (n = 2). One dog was mildly hypercalcemic at baseline (11.9 mg/dL), and remained mildly hypercalcemic during the calcifediol supplementation. There was no observed association between serum tCa and 25(OH)D concentrations (*P* = .52).

No dog developed hyperphosphatemia based on our laboratory's reference range (ie, >8.1 mg/dL); however, this upper limit of the reference range is not consistent with optimal phosphorus control (ie, serum phosphorus <4.6 mg/dL), as recommended by the IRIS CKD Treatment Guidelines. Six dogs demonstrated a serum phosphorus ≥4.6 mg/dL during the course of the study. Two dogs had a serum phosphorus >4.6 mg/dL at baseline (5.7 mg/dL and 6.5 mg/dL), and remained >4.6 mg/dL during calcifediol supplementation (up to 7.0 mg/dL). Three dogs had mild isolated increases in phosphorus during calcifediol supplementation at day 28 (n = 1), day 54 (n = 1) and day 84 (n = 2). There was no observed association between serum phosphorus and 25(OH)D concentrations (*P* = .17). There were no observed differences in CaPP over the duration of dosing (*P* = .22).

There were no observed differences in UCa/Cr over the duration of the dosing (*P* = .14). Fractional excretion of tCa, iCa, phosphorus, and sodium were measured and no observed differences were noted over the duration of dosing (Table 1).

### 3.4 Parathyroid hormone and fibroblast growth factor‐23

**FIGURE 4:**
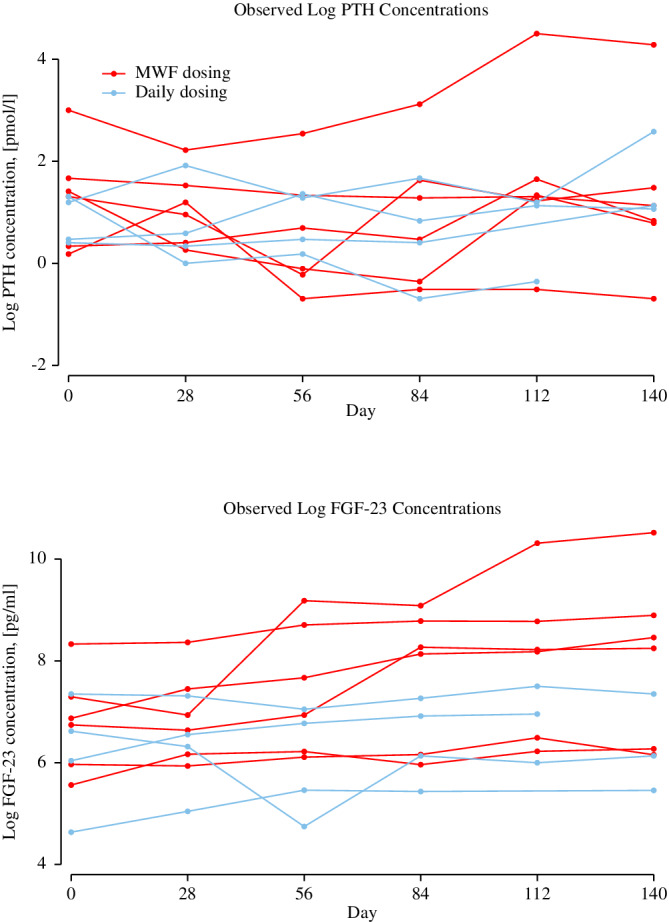
Serum PTH and plasma FGF‐23 concentrations at baseline, during 84 days of extended‐release calcifediol supplementation, and after discontinuation of calcifediol supplementation. Each line plot represented observed values from an individual dog. Dogs receiving daily dosing are graphed in blue, and dogs receiving intermittent dosing on Monday, Wednesday, and Friday each week (M‐W‐F dosing) are graphed in red

Serum PTH and plasma FGF‐23 concentrations are listed in Table 1. There were no observed differences in PTH concentrations at any timepoint from baseline (*P* = .26). For FGF‐23 concentrations, differences were observed at days 84, 112, and 140 (*P* < .01 for all time‐points). This remained true even when 1 dog whose FGF‐23 concentrations increased much more than all others was removed from the model (Figure 4). There was no observed association between FGF‐23 concentrations and FE of phosphorus (*P* = .64).

### 3.5 Diet, medications, and supplements

All 10 dogs were eating a veterinary therapeutic renal diet, albeit from a variety of manufacturers, and received a variety of treats. Concomitant medications administered during the study included gabapentin (n = 5), maropitant [n = 5; (1 dose only n = 4)], aluminum hydroxide (n = 3), trazodone (n = 3), enalapril (n = 2), omeprazole (n = 2), ondansetron (n = 2), phenylpropanolamine (n = 2), amlodipine (n = 1), aspirin (n = 1), carvedilol (n = 1), diphenhydramine (n = 1), hydroxyzine (n = 1), fluoxetine (n = 1), mirtazapine (n = 1) mycophenolate mofetil (n = 1), ophthalmic drops (n = 2), telmisartan (n = 1), tramadol (n = 1), tylosin (n = 1). Monthly heartworm and flea and tick preventatives were provided to 6 dogs. Supplements that dogs received included glucosamine (n = 5), psyllium (n = 4), probiotic (n = 2), fish oil (n = 1), flax seed oil (n = 1), ocular supplement (n = 1; Ocuvite, Baush + Lomb, Bridgewater, New Jersey) tryptophan supplement (n = 1), turmeric (n = 1).

### 3.6 Quality of life questionnaire

**FIGURE 5:**
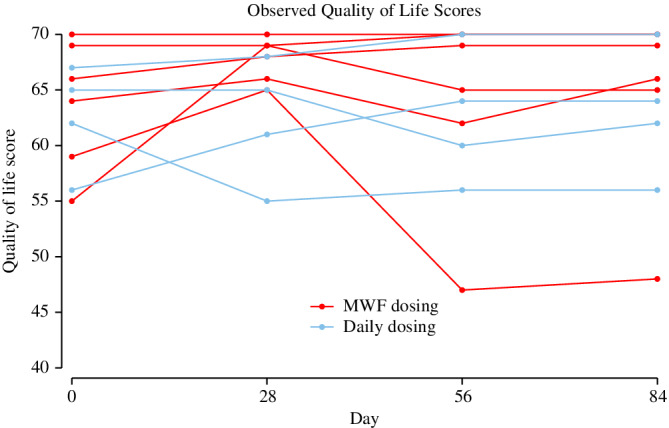
Quality of life (QOL) scores for each dog (n = 10) at baseline and during 84 days of extended‐release calcifediol supplementation. Each line plot represented observed values from an individual dog. Dogs receiving daily dosing are graphed in blue, and dogs receiving intermittent dosing on Monday, Wednesday, and Friday each week (M‐W‐F dosing) are graphed in red

At baseline, the median QOL score was 64.5 (range, 55‐70). There were no observed differences in QOL scores over time during the study duration (*P* = .52). In 8 dogs, QOL scores either remained static or increased over the 84 day calcifediol supplementation period. Two dogs had diminishing QOL scores (Figure 5). There was no observed association between QOL score and serum creatinine (*P* = .34) nor serum 25(OH)D (*P* = .51).

## 4 DISCUSSION

In this study, calcifediol supplementation increased serum concentrations of 25(OH)D, 1,25(OH)_2_D, and 24,25(OH)_2_D over baseline by a median of 470%, 94%, and 321% at day 84, respectively. The greatest increase in serum concentrations was observed between baseline and day 28, and serum concentrations continued to increase until the calcifediol supplement was discontinued at day 84. Vitamin D metabolites are highly correlated variables, and this is reflected in the results as supplementation with calcifediol significantly increased all measured vitamin D metabolites.

The optimal goal for serum 25(OH)D concentration during vitamin D supplementation in dogs is currently unknown. One study suggested that a serum concentration of 100 ng/mL is optimal, based on a plateau of serum ionized PTH concentrations in healthy dogs and dogs with hemoabdomen.^25^ Despite some individual laboratories having reference ranges for normal dogs, there are no well‐defined physiologic reference ranges for “normal” 25(OH)D concentrations nor for insufficiency or deficiency. In 1 study, the range of serum 25(OH)D concentrations in healthy dogs ranged from 9.5 to 249.2 ng/dL, indicating that there is tremendous variability even in a healthy dog population.^26^ In people, vitamin D insufficiency is currently defined as a serum 25(OH)D concentration <30 ng/mL, and goal of vitamin D supplementation is typically to increase 25(OH)D above 30 ng/mL.^27^ However, human reference ranges cannot be applied to dogs as there are significant differences in dietary intake of vitamin D, calcium and phosphorus between the species as well as baseline serum concentrations in otherwise healthy individuals.

One of the unique properties about this extended‐release calcifediol medication in people is that gradual increases in serum concentrations of 25(OH)D develop. This gradual increase is thought to have minimal effect on upregulation of the gene responsible for 24‐hydroxylase (24[OH]ase) activity (CYP24A1) that otherwise occur during a rapid increase of 25(OH)D. This leads to reduced catabolism of 25(OH)D to 24,25(OH)_2_D, and potentially has the indirect effect of increasing conversion of 25(OH)D to 1,25(OH)_2_D (calcitriol) as more substrate is presented to the kidneys.^28^ It has recently been proposed that serum 24,25(OH)_2_D concentration might serve as a reliable indicator of serum 25(OH)D concentrations, and that the ratio of 25(OH)D to 24,25(OH)_2_D might help predict relative 24(OH)ase activity, with decreased ratios indicating increased 24(OH)ase activity and possible resistance to vitamin D treatment.^29^ In this study, there were no differences in this ratio over the course of the study. The authors cannot comment on how a nonextended‐release calcifediol supplement would perform in dogs with CKD.

In people, overt toxicosis with vitamin D supplementation, associated with development of hypercalcemia, is thought to occur when 25(OH)D concentrations exceed 150 to 200 ng/mL. In various animal species (rats, cows, pigs, rabbits, dogs, and horses), plasma 25(OH)D concentrations associated with hypercalcemia have exceeded 150 ng/mL.^30^ It is likely that the rate of rise in 25(OH)D as well as the specific supplement provided will influence the likelihood of overt toxicity. Dogs in this study had serum 25(OH)D concentrations >150 ng/mL for most of the study period and did not exhibit overt clinical signs of vitamin D toxicity.

While some dogs did develop mild total hypercalcemia during calcifediol supplementation, there was no correlation between serum tCa and 25(OH)D concentrations. Discordance between total calcium and iCa occurs in some dogs with CKD, likely due to increases in the complexed fraction of tCa that can occur.^31^ It is possible that the oral 25(OH)D supplementation did affect intestinal absorption of calcium in these dogs, as 25(OH)D consumption results in upregulation of the VDR in the duodenum of mice.^32^ The amount of calcium in the different veterinary therapeutic renal diets consumed by dogs in the study ranged from approximately 0.73 g per 1000 kcal (Mcal) to 1.95 g per Mcal.

Urine Ca/Cr ratios were measured to monitor for early signs of hypervitaminosis D as this can be an earlier indicator than hypercalcemia.^33^ While there are not fully established normal reference ranges for UCa/Cr in dogs, 1 study used 0.05 as a cut‐off to document hypercalciuria; this was based on the upper end of the 95% confidence interval for control dogs.^34^ In our study, there was not a significant increase in median UCa/Cr with calcifediol supplementation. One dog did have an increase from baseline to day 84, from 0.01 to 0.07. This dog received daily calcifediol supplementation and ultimately had the highest serum 25(OH)D concentration documented at 469.9 ng/dL. One dog had a higher baseline UCa/Cr of 0.07 with subsequent values of 0.12, 0.13, and 0.11 at days 28, 56, and 84, respectively. This dog was a 1‐year‐old large breed dog, and its age might have contributed to higher UCa/Cr ratios, as UCa/Cr ratios vary with age in children.^35^

Despite a lack of significant increase in UCa/Cr, there was an increase in FE of calcium while dogs were receiving the extended‐release calcifediol supplement. In 1 study of 17 normal dogs of various breeds, the range of FE of calcium was 0.053%‐0.555%.^36^ In another study of 48 Greyhound dogs, the range of FE of calcium was 0.03%‐0.22%.^36^ Both of these studies entered tCa into the equation.^36^, ^37^ In our study, median FE of calcium, based on tCa, increased from 0.18% to 0.51% after 84 days of calcifediol treatment. Using iCa in the equation resulted in a larger calculated number since iCa is of lower magnitude than tCa by about 50%. Neither the use of tCa nor iCa is ideal for the calculation of FE of calcium, since neither fully accounts for the ultrafilterable fraction of calcium (ionized plus complexed fractions that cross the glomerulus). Use of tCa overestimates the amount of calcium available for glomerular filtration since it includes the protein‐bound fraction which does not enter urine in the absence of severe proteinuria. Serum iCa underestimates how much calcium is available for filtration since it does include the complexed fraction of calcium that also crosses the glomerulus.

The significance for the increased FE of calcium in this study and possible long‐term adverse effects remain unknown. No short‐term adverse effects were identified. Theoretically future studies should evaluate dogs for the presence of calcium‐related crystalluria and stones. It has been reported that FE of all electrolytes increase during CKD, and FE is not expected to match up with the absolute quantity for total daily excretion of electrolytes in CKD (mg or mEq).^21^, ^38^, ^39^ Normal FE of phosphorus in dogs has been reported to range from 11%‐41%,^36^, ^37^ which was similar to our results. There was no correlation between FE of phosphorus and plasma FGF‐23.

Serum PTH concentration did not decrease after calcifediol supplementation as we had expected. Increased circulating 25(OH)D and 1,25(OH)_2_D concentrations are associated with downregulation of PTH synthesis and secretion in human CKD. Alternatively, the failure of PTH to progressively increase over several months in dogs with CKD could indicate vitamin D metabolite suppression of higher levels of circulating PTH, but this cannot be determined without comparison to a control group without treatment.

Fibroblast growth factor‐23 concentrations increased from baseline to day 84, and remained increased through day 140. There are a couple of possible explanations for this observation. It is possible that this was simply a matter of time and disease progression.^23^ Serum phosphorus concentrations did not significantly increase over time, and it is possible that the increased FGF‐23 concentrations helped control hyperphosphatemia. It is unknown how FGF‐23 and calcitriol concentrations might have affected one another. Calcitriol concentrations most likely increased due to increased synthesis from very high concentrations of serum 25(OH)D presented as substrate, and possibly due to extrarenal 1α‐hydroxylase activity. Calcitriol is expected to stimulate increased synthesis and secretion of FGF‐23 from osteocytes, and then FGF‐23 downregulates 1α‐hydroxylase activity. This raises an additional question about how to balance the goal of increasing calcitriol to positively impact cellular health while not adversely increasing FGF‐23 concentrations.

There were a few limitations of this study. It would have been ideal to obtain more complete data sets at days 112 and 140 (eg, serum iCa concentrations, UCa/Cr). This was a short pilot study primarily designed to determine (a) efficacy of serum 25(OH)D repletion and (b) tolerance of the calcifediol supplement. This study was unable to specifically determine the optimal dose of extended‐release calcifediol supplementation. The data generated did not determine if, and to what extent, the peak of 25(OH)D concentrations would have continued increasing in dogs with either daily or 3 times weekly dosing. Dogs that received the supplement 3 times weekly had a lower peak of serum 25(OH)D than dogs that received daily supplementation, and there was no evidence of toxicity with this dose.

Sample size is an important consideration in any study, and this might have affected the results in certain aspects of this study. One of the goals of this study was to obtain an estimate of standard deviation to plan further studies in this area of research. This study consisted of 10 dogs based on both preclinical data from the drug manufacturer and budget considerations. Specifically, the relatively small study size might limit our ability to detect some true differences and associations in this study population. Further comprehensive studies in larger cohorts are needed to confirm or refute these results based on the data and effect sizes of the variables presented here.

## CONFLICT OF INTEREST DECLARATION

Dr. Beizaei is employed by EirGen Pharma, a subsidiary of OPKO Health, Inc. Drs. Parker and Chew are in discussions for research projects with Dr Beizaei and EirGen Pharma.

## OFF‐LABEL ANTIMICROBIAL DECLARATION

Authors declare no off‐label use of antimicrobials.

## INSTITUTIONAL ANIMAL CARE AND USE COMMITTEE (IACUC) OR OTHER APPROVAL DECLARATION

Approved by The Ohio State University IACUC.

## HUMAN ETHICS APPROVAL DECLARATION

Authors declare human ethics approval was not needed for this study.

## Supporting information


**Data S1** Appendix: Quality of life (QOL) questionnaire.
