# Supplementary material for: Effects of calcifediol supplementation on markers of chronic kidney disease‐mineral and bone disorder in dogs with chronic kidney disease
Source: J Vet Intern Med. 2020 Oct 31;34(6):2497–506. doi: 10.1111/jvim.15949 (PMC7694821; doi:10.1111/jvim.15949)
Supplement: Supplementary file 1 — Data S1 Appendix: Quality of life (QOL) questionnaire. [file JVIM-34-2497-s001.pdf]

Canine Kidney Disease Treatment Survey

Survey Date_________________

Name of person completing survey________________________________

Pet name__________________________

****

Instructions: Please indicate your assessment by circling the number on the scale next to each question, providing your opinion on your pet's CURRENT health status.

1 = strongly disagree 2 = disagree 3 = neutral 4 = agree 5 = strongly agree

****

**Happiness**					
My dog responds to my presence	1	2	3	4	5
My dog enjoys life	1	2	3	4	5
**Mental status**					
My dog has more good days than bad days	1	2	3	4	5
My dog seems alert, <u>not</u> dull or depressed	1	2	3	4	5
**Appetite & gastrointestinal health**					
My dog enjoys eating	1	2	3	4	5
My dog eats the recommended amount of food daily	1	2	3	4	5
My dog eats the same food every day	1	2	3	4	5
My dog does <u>not</u> act nauseous or vomit	1	2	3	4	5
My dog has normal stool quality	1	2	3	4	5
**Mobility & recreation**					
My dog moves normally	1	2	3	4	5
My dog enjoys playing	1	2	3	4	5
My dog is as active as he/she has been	1	2	3	4	5
**General health**					
My dog's overall health is the same as compared to his/her last evaluation	1	2	3	4	5
My dog's overall health is the same as compared to initial diagnosis of kidney disease	1	2	3	4	5

Owners will also fill out daily activity diaries.
